# Antismoking Advertisements and Price Promotions and Their Association With the Urge to Smoke and Purchases in a Virtual Convenience Store: Randomized Experiment

**DOI:** 10.2196/14143

**Published:** 2019-10-23

**Authors:** Lauren McCarl Dutra, James Nonnemaker, Brian Bradfield, Nathaniel Taylor, Jamie Guillory, Ashley Feld, Annice Kim

**Affiliations:** 1 Center for Health Policy Science and Tobacco Research RTI International Berkeley, CA United States; 2 Center for Health Policy Science and Tobacco Research RTI International Research Triangle Park, NC United States; 3 Prime Affect Research Dublin Ireland

**Keywords:** cigarette smoking, advertisement, craving, tobacco products, commerce, consumer behavior

## Abstract

**Background:**

Point of sale (POS) advertising is associated with smoking initiation, current smoking, and relapse among former smokers. Price promotion bans and antismoking advertisements (ads) are 2 possible interventions for combating POS advertising.

**Objective:**

The purpose of this analysis was to determine the influence of antismoking ads and promotions on urges to smoke and tobacco purchases.

**Methods:**

This analysis examined exposure to graphic (graphic images depicting physical consequences of tobacco use) and supportive (pictures of and supportive messages from former smokers) antismoking ads and promotions in a virtual convenience store as predictors of urge to smoke and buying tobacco products among 1200 current cigarette smokers and 800 recent quitters recruited via a Web-based panel (analytical n=1970). We constructed linear regression models for urge to smoke and logistic regression models for the odds of purchasing tobacco products, stratified by smoking status.

**Results:**

The only significant finding was a significant negative relationship between exposure to supportive antismoking ads and urge to smoke among current smokers (beta coefficient=−5.04, 95% CI −9.85 to −0.22; *P*=.04). There was no significant relationship between graphic antismoking ads and urge to smoke among current smokers (coefficient=−3.77, 95% CI −8.56 to 1.02; *P*=.12). Neither relationship was significant for recent quitters (graphic: coefficient=−3.42, 95% CI −8.65 to 1.81; *P*=.15 or supportive: coefficient=−3.82, 95% CI −8.99 to 1.36; *P*=.20). There were no significant differences in urge to smoke by exposure to promotions for current smokers (coefficient=−1.06, 95% CI −4.53 to 2.41; *P*=.55) or recent quitters (coefficient=1.76, 95% CI −2.07 to 5.59; *P*=.37). There were also no differences in tobacco purchases by exposure to graphic (current smokers: coefficient=0.93, 95% CI 0.67 to 1.29; *P*=.66 and recent quitters: coefficient=0.73, 95% CI 0.44 to 1.19; *P*=.20) or supportive (current smokers: coefficient=1.05, 95% CI 0.75 to 1.46; *P*=.78 and recent quitters: coefficient=0.73, 95% CI 0.45 to 1.18; *P*=.20) antismoking ads or price promotions (current smokers: coefficient=1.09, 95% CI 0.86 to 1.38; *P*=.49 and recent quitters: coefficient=0.90, 95% CI 0.62 to 1.31; *P*=.60).

**Conclusions:**

The results of this analysis support future research on the ability of supportive antismoking ads to reduce urges to smoke among current cigarette smokers. Research on urges to smoke has important tobacco control implications, given the relationship between urge to smoke and smoking cigarettes, time to next smoke, and amount smoked.

## Introduction

### Background

Tobacco advertising promotes tobacco use, which results in 480,000 deaths each year in the United States [1]. In 2015, tobacco companies spent approximately US $8.5 billion, or 95% of their advertising budget, on the tobacco retail environment, otherwise known as the point of sale (POS) [2]. POS marketing influences susceptibility to smoking among youth [3,4] and quitting behavior among adults [5,6].

Moreover, 2 common and effective POS marketing techniques are tobacco displays (ie, large, colorful displays of tobacco products often referred to as power walls [7]) and price promotions, such as coupons and multipack discounts [8]. Tobacco displays and other forms of protobacco advertising at the POS have been associated with cravings to smoke among current and former tobacco users [9]. Tobacco displays are also associated with susceptibility to smoking among youth, fewer successful quit attempts among adults, and unplanned purchases of tobacco products among tobacco users [3,5,6,10-12].

Promotions are used by tobacco companies to offset price increases caused by tobacco control policies, such as taxes [13]. Promotions have been associated with current smoking among youth [4] and purchasing larger quantities of cigarettes per store visit among adult smokers [14]. Researchers found that New York State counties with a greater number of retail cigarette promotions between 2004 and 2008 also had a higher youth smoking prevalence [4]. Similarly, another study of combustible tobacco users living in the rural United States found that those who used promotions purchased more cigarettes [14].

Furthermore, 1 potential method of counteracting the effects of tobacco displays and promotions is antitobacco ads [9]. These ads, particularly those with emotional components (such as personal stories and graphic images), have been associated with higher odds of quitting smoking among US adult smokers [15]. In 2009, New York City posted graphic antismoking warning signs in tobacco retail stores [9]. After the warning signs were posted, visitors to New York City retail stores who viewed the warning signs (and protobacco advertising) were significantly more likely to report that the signs made them think about the health risks of smoking or quitting smoking compared with those who visited the stores before the warning signs were posted [9]. Similarly, graphic warning labels on cigarette packs have been associated with lower cravings to smoke [16].

However, 1 study suggests that antismoking ads (referred to hereafter as antismoking ads) may not be effective [17,18]. In a virtual shopping experiment [17], researchers exposed adult current cigarette smokers and recent quitters to either a closed tobacco display with no advertising or a closed display with a graphic antismoking ad. The researchers did not find any differences in urge to smoke or purchase attempts based on exposure to the antismoking ads. The US Food and Drug Administration implemented the first national POS antismoking media campaign, Every Try Counts [18], in 2018. The campaign targets adult cigarette smokers, particularly those who are trying to quit smoking cigarettes despite multiple failed quit attempts. The campaign involves placing supportive antismoking ads that depict former smokers who appear to be in good health, and the ads contain prosmoking cessation messages such as If at first you don’t succeed, try, try again. These messages are designed to promote quit attempts among adult cigarette smokers in convenience stores around the United States. Evaluation of the campaign is currently underway.

Another approach to countering protobacco advertising at the POS is banning price promotions. These bans prevent retailers from discounting tobacco products (such as buy 1 get 1 free or US $1 off) and, therefore, have the potential to influence consumer tobacco purchases [19]. Several counties and US states have enacted price promotion bans. As of March 2018, 13 local governments in Massachusetts (Chelsea and Winthrop), Michigan (East Lansing), Minnesota (North Branch and Wyoming), New York (New York City), Rhode Island (Providence and Central Falls), Texas (Rockport and Magnolia), and Washington (Cheney, Spokane, and Millwood) had passed regulations to counteract price promotions on tobacco products. These areas passed minimum prices on cigarette packs, prohibited or restricted the ability of retailers to redeem coupons or use price-reduction promotions (eg, multipack discounts), and set a minimum pack size and prices for tobacco products other than cigarettes (eg, cigarillos and cigars) [20].

### Objectives

To further understand the potential effects of antismoking ads, price promotions, and their combined effect on urges to smoke and tobacco purchases, we used RTI iShoppe (iShoppe), a virtual convenience store developed by RTI International, to conduct an experiment that used a virtual convenience store to vary these aspects of the retail environment. Virtual stores, which simulate a retail shopping experience, are helpful for evaluating the effects of new or potential tobacco control approaches. We created different versions of convenience stores in iShoppe to test the effects of antismoking ads (graphic ads, supportive ads, or no ads) and price promotions (present vs absent) on urges to smoke and tobacco purchases among current and former cigarette smokers. Graphic ads contain graphic depictions of the physical consequences of tobacco use. Supportive ads include pictures of and supportive messages from former smokers who appear healthy. This research has the potential to contribute to the existing evidence base for the use of antismoking ads and price promotion bans (as well as a combination of the 2) as tobacco control measures at the POS.

Given the evidence establishing a relationship between warning images and cravings to smoke [16], we hypothesized that participants exposed to antismoking ads would report lower urges to smoke and purchase fewer tobacco products in iShoppe. We also hypothesized that participants exposed to price promotions would report greater urges to smoke and be more likely to purchase tobacco products than those who were not exposed to promotions. In addition, given the differences in responses to advertising by smoking status [21-23], we hypothesized that smoking status would serve as an effect modifier of the relationship between the tobacco control measures and outcomes examined in this analysis.

## Methods

### Participants

We used Lightspeed’s Web-based survey panel to recruit a national convenience sample of 1200 adult current cigarette smokers and 800 recent quitters. First, potential participants completed a screening survey to ensure that they met the inclusion criteria. Current cigarette smokers or recent quitters older than 18 years were eligible to participate. Current smokers were participants who had smoked at least 100 cigarettes in their lifetimes and who reported that they currently smoked every day or some days. Recent quitters were participants who had smoked at least 100 cigarettes in their lifetimes, were now smoking not at all, and reported that they had quit smoking within the past year.

### Study Procedures

iShoppe is a 3-dimensional (3D) virtual environment based on an off-the-shelf model of a convenience store that was extensively customized using the Unity 3D interaction gaming software. Since the original version of the virtual store [24], which was based on feedback from focus groups, the store has been updated many times.

For this study, Lightspeed provided participants with a link to access the store. If the participant already had the Unity 3D player installed, the store loaded once the participant clicked on the link. If it was not installed, participants were provided with instructions on how to download it. The first screen displayed contained a list of instructions on how to use the store, including the keystrokes used to explore the store. Each participant was provided a budget of US $15 or US $20 for the experiment. In areas with higher tobacco product prices, a US $20 budget was given to ensure all participants could purchase tobacco products. Participants were informed of their budget and instructed to purchase whatever they wanted to purchase in the store (within the budget). Participants were provided instructions for completing their purchases and were given 10 min to complete the shopping task. Further information about iShoppe is available from previous publications [4,17,24-26]. After completing the shopping task, participants were routed to a survey that measured urge to smoke, recall of products and ads in the virtual store, usual tobacco purchasing behavior, and tobacco use.

### Experimental Design

**Figure 1:**
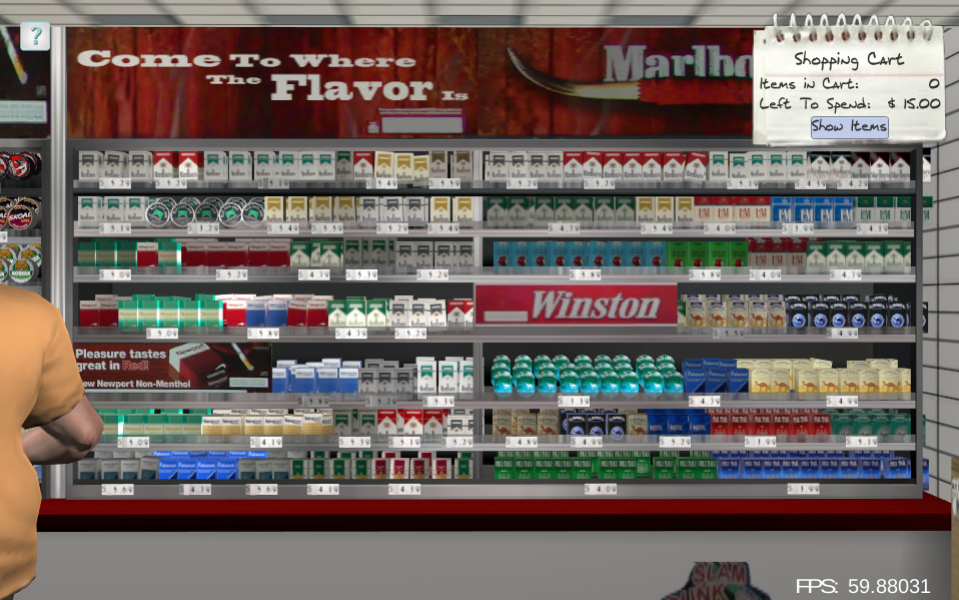
Condition 1: Antismoking advertisements are absent, and price promotions are banned.

**Figure 2:**
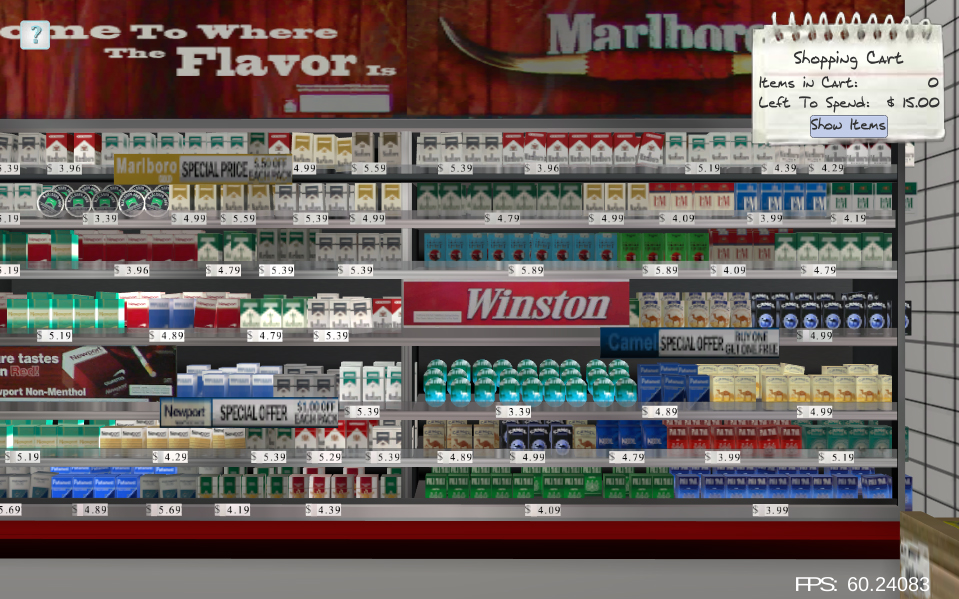
Condition 4: Graphic advertisements (ads) are present in purchasable ad space (eg, interior and exterior windows, gas pump topper) and price promotions are present (text on poster on pillar reads "SPECIAL OFFER: Buy two packs, get one free").

**Figure 3:**
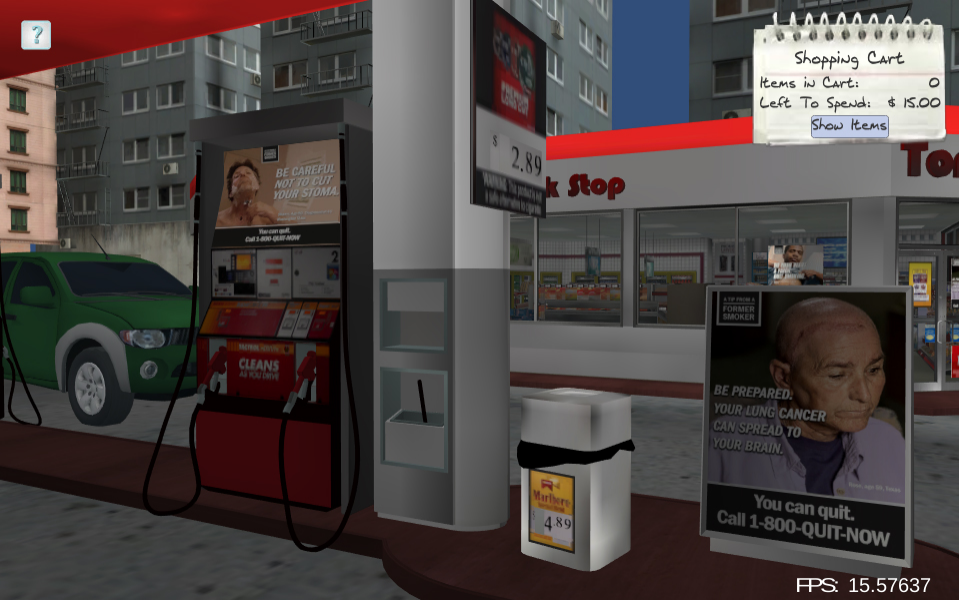
Condition 5: Graphic advertisements (ads) are present in purchasable (eg, interior and exterior windows, gas pump topper) and high visibility (eg, by checkout counter, hanging from the ceiling between aisles) ad space, and price promotions are banned.

**Figure 4:**
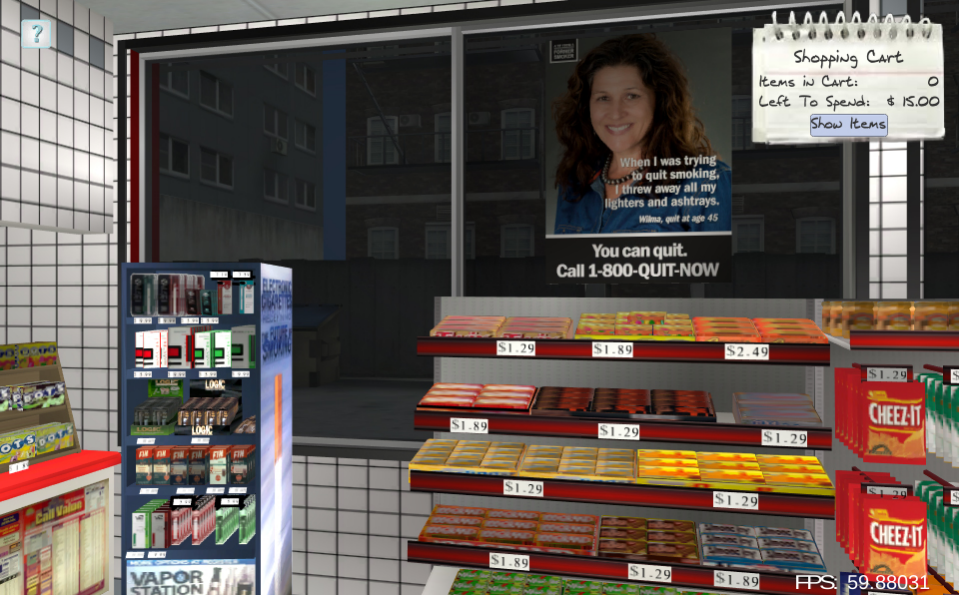
Condition 7: Supportive advertisements (ads) are present in purchasable ad space (eg, interior and exterior windows, gas pump topper), and price promotions are banned.

The study used a partially crossed 3 (antismoking ad type: graphic, supportive, or none) by 2 (antismoking ad placement: purchasable ad space only or purchasable ad space plus high-visibility ad space) by 2 (promotions: absent or present) design. Study conditions with no antismoking ads did not include variation by ad placement, making the design partially crossed. The experimental design contained 10 conditions (Figures 1-4, Multimedia Appendices 1 and 2). Approximately 200 participants were assigned to each condition (ie, 1200 current smokers and 800 recent quitters). However, this analysis focuses on the effects of antismoking ads and price promotions, but not placement of antismoking ads, because we were most interested in the main effects of antismoking ads and promotions. As a result, when we conducted the analysis, we collapsed conditions 3 and 5 (graphic ads and promotions banned [Figure 3]), 7 and 9 (supportive ads and promotions banned), 4 and 6 (graphic ads and promotions present), and 8 and 10 (supportive ads [Figure 4] and promotions present). Therefore, this analysis contains 2 variables (ad placement not included) and 6 conditions (Multimedia Appendix 3).

### Variables

#### Outcome Variables

Urge to smoke was assessed immediately after completing the virtual store shopping task, on a scale from 0 to 100, with 0 indicating no urge and 100 indicating the strongest urge I have ever experienced. Purchasing 1 or more tobacco products of any kind (by clicking to purchase) was also an outcome variable. The tobacco products available for purchase in the virtual store were cigarettes, cigars (including cigarillos and little cigars), smokeless tobacco, and electronic cigarettes (e-cigarettes).

#### Independent Variable

The exposure variables were antismoking ad condition (graphic, supportive, or neither, the last of which is the reference category) and price promotion condition (present or absent, the latter of which is the reference category).

#### Antismoking Advertisement Condition

We used antismoking ads from Centers for Disease Control and Prevention’s (CDC) *Tips from Former Smokers* (*Tips)* national campaign. We chose these ads because they included graphic and supportive messages but were otherwise similar. We removed the CDC logo and placed national Quitline information (You can quit. Call 1-800-QUIT-NOW) at the bottom of all ads for consistency. Antismoking ads were placed in locations outside (gas pump toppers, walls, sandwich boards, ice chests, windows, and store entrance doors) and inside (interior windows and drink coolers) of the store that are typically available for purchase in most convenience stores. In the high-visibility condition, antismoking ads were also placed above the checkout counter (overhang) and hung from the ceiling in each center aisle.

#### Price Promotion Condition

The price promotion condition included special prices on specific items (eg, special price: US $0.50 off each pack) and discounts for buying more than 1 of the same product type (ie, multipack discounts). Promotions were placed on the tobacco display and ad posters for leading brands of specific tobacco products, including cigarettes, little cigars or cigarillos, smokeless tobacco, and e-cigarettes. The prices on the ads were customized based on each participant’s geographic location to reflect the tobacco product pricing and regulations in each state. In all conditions, the store contained a visible tobacco display behind the checkout counter and ads for tobacco products.

#### Covariates

Covariates included age (18-24 [reference category], 25-34, 35-54, and ≥55 years), gender (male [reference category] or female), race and ethnicity (non-Hispanic white [reference category], non-Hispanic black, Hispanic, or non-Hispanic, other race), education (less than high school, high school graduate, some college, or a college degree or greater [reference category]), and the frequency at which the participant visited convenience stores (coded as a range from hardly ever [1] to daily [5]). We also examined the number of days smoked in the past 30 days among current smokers.

### Statistical Analysis

We excluded participants with missing values for our outcome variables of interest, resulting in an analytic sample of 1970 participants (98.50% of the original sample of 2000 participants).

#### Descriptive Statistics

We tested for an imbalance in demographic covariates across conditions, which can sometimes occur even with randomization. Demographic covariates were included in multivariable models if they varied by experimental condition at the *P*<.10 level [27].

#### Bivariate Statistics

Next, we conducted bivariate analyses to understand the relationship between antismoking ads, price promotions, and the outcome variables using *t* tests for urge to smoke by price promotion; a 1-way analysis of variance for urge to smoke by antismoking ad condition; and chi-square analyses for tobacco purchases by promotion and antismoking ad condition.

#### Interactions

Then, to assess the interaction effects of the 2 independent variables, we tested interaction terms for antismoking ad by price promotions. Interaction terms that were significant at the *P*<.10 level were included in final regression models.

#### Covariate-Adjusted Regression Models

Finally, we constructed linear regression models for urge to smoke and logistic regression models for tobacco purchases. As we hypothesized that there would be differences in reactions to the store environment based on smoking status, we stratified all models by smoking status. We included all covariates in regression models that varied at the *P*<.10 level, with the exception of days smoked in the past 30, which we did not include because the variable was only relevant for current smokers (not for recent quitters).

## Results

### Descriptive Statistics

More than half of participants were current smokers (1177/1970, 59.75%); 40.25% (793/1970) of the participants were recent quitters (Table 1). The largest age group included participants aged from 35 to 54 years (636/1970, 32.28%), and the majority of the sample was female (1349/1970, 68.48%). The sample was primarily non-Hispanic white (1581/1970, 77.04%), and almost half of the sample (859/1970, 43.62%) had a college degree or greater education. On average, current smokers in the sample reported smoking on 25.6 (SD 8.2) of the past 30 days.

**Table 1:** Demographics characteristics of participants in the RTI iShoppe antismoking ad and price promotion study (n=1970).

Characteristics		Values
**Smoking status, n (%)**		
	Current smoker	1177 (59.75)
	Recent quitter	793 (40.25)
**Age (years), n (%)**		
	18-24	271 (13.76)
	25-34	519 (26.35)
	35-54	636 (32.28)
	≥55	544 (27.61)
**Female, n (%)**		1349 (68.46)
**Race, n (%)**		
	Non-Hispanic white	1518 (77.04)
	Non-Hispanic black	111 (5.64)
	Hispanic	189 (9.58)
	Non-Hispanic other	152 (7.74)
**Education, n (%)**		
	Less than high school	42 (2.14)
	High school graduate or General Educational Development degree	428 (21.71)
	Some college	640 (32.54)
	College graduate and beyond	859 (43.62)
**Days smoked in the past 30 (current smokers only), mean (SD)**		25.6 (8.2)

As expected, because of randomization, there were few differences in demographic characteristics between conditions. A greater proportion of participants in the antismoking ad condition (513/1579, 32.49%) reported attending some college compared with those in the no antismoking ad condition (111/391, 28.5%; *P*=.049). The antismoking ad condition also had fewer white participants (1146/1579, 76.2%) than the no antismoking ad condition (314/391, 80.2%; *P*=.08). Owing to these differences, race and ethnicity and education were included in all multivariable models.

### Bivariate Statistics

#### Tobacco Purchases

There was no difference in tobacco purchases by antismoking ad condition (*P*=.78) or price promotion condition (*P*=.87; Table 2).

#### Urge to Smoke

Bivariate analyses of the outcome variables by antismoking ad (Table 2) revealed a lower urge to smoke among participants who viewed antismoking ads (at the *P*<.10 level [*P*=.08]). However, there was no difference in urge to smoke by presence of a price promotion (*P*=.58).

**Table 2:** In-store behaviors and self-reported urge to smoke cigarettes by smoking status among participants in the RTI iShoppe antismoking ad and price promotion study (n=1970).

Variable	Antismoking ad condition				Price promotions		
	Graphic (n=789)	Supportive (n=790)	Neither (n=391)	*P* value	Banned (n=981)	Present (n=989)	*P* value
Purchased any tobacco, n (%)	338 (42.8)	345 (43.7)	176 (45.01)	.78	426 (43.4)	433 (43.8)	.87
Urge to smoke (1-100), mean (SE)	42.1 (1.2)	40.3 (1.1)	44.8 (1.6)	.08	41.8 (1.1)	42.1 (1.0)	.58

### Interactions

None of the interaction terms tested was significant at the *P*<.10 level for urge to smoke (current smokers: *P*=.25 for graphic ads by promotions and *P*=.91 for supportive ads by promotions; recent quitters: *P*=.93 for graphic ads by promotions and *P*=.21 for supportive ads by promotions), and therefore, the models of urge to smoke did not contain interaction terms. In the models of tobacco purchases, *P* values were .08 (graphic) and .27 (supportive) for interactions for current smokers and .34 (graphic) and .65 (supportive) among recent quitters. We did not include interaction terms for current smokers because the results of the model remained virtually the same (graphic antismoking ad: odds ratio [OR]=0.09, 95% CI 0.45 to 1.76; supportive antismoking ad: OR=0.65, 95% CI 0.33 to 1.30; promotion: OR=0.99, 95% CI 0.46 to 2.12).

### Covariate-Adjusted Regression Models

#### Urge to Smoke

Adjusting for covariates, current smokers exposed to supportive antismoking ads (coefficient=–5.04, 95% CI −9.85 to −0.22; *P*=.04; Table 3) reported significantly lower urges to smoke after visiting iShoppe than current smokers who were not exposed to the antismoking ads. However, there was no difference in urge to smoke based on exposure to graphic ads (coefficient=–3.77, 95% CI −8.56 to 1.02; *P*=.12) among current smokers (Table 3).

Among recent quitters, there was no difference in urge to smoke between those who did and did not see antismoking ads (graphic: coefficient=−3.42, 95% CI −8.65 to 1.81; *P*=.20 and supportive: coefficient=−3.82, 95% CI −8.99 to 1.36; *P*=.15).

There were no significant differences in urge to smoke between participants exposed to price promotions (vs not exposed) among current smokers (coefficient=−1.06, 95% CI −4.53 to 2.41; *P*=.55) or recent quitters (coefficient=1.76, 95% CI −2.07 to 5.59; *P=*.37).

**Table 3:** Multivariable regression models of urge to smoke (linear) and tobacco purchases (logistic) regressed on antismoking ad condition and presence of price promotions in the RTI iShoppe virtual convenience store (n=1970).

Exposure variable name, categories		Urge to smoke		Bought tobacco	
		Current smokers, beta coefficient (95% CI)	Recent quitters, beta coefficient (95% CI)	Current smokers, odds ratio (95% CI)	Recent quitters, odds ratio (95% CI)
**Antismoking ad condition**					
	Graphic	−3.77 (−8.56 to 1.02)	−3.42 (−8.65 to 1.81)	0.93 (0.67 to 1.29)	0.73 (0.44 to 1.19)
	Supportive	−5.04 (−9.85 to −0.22)	−3.82 (−8.99 to 1.36)	1.05 (0.75 to 1.46)	0.73 (0.45 to 1.18)
	Neither	Reference	Reference	Reference	Reference
**Price promotions**					
	Banned	Reference	Reference	Reference	Reference
	Present	−1.06 (−4.53 to 2.41)	1.76 (−2.07 to 5.59)	1.09 (0.86 to 1.38)	0.90 (0.62 to 1.31)
**Race**					
	Non-Hispanic white	Reference	Reference	Reference	Reference
	Non-Hispanic black	5.23 (−2.37 to 12.83)	−5.25 (−13.58 to 3.09)	0.58 (0.35 to 0.96)	0.80 (0.33 to 1.94)
	Hispanic	11.80 (5.71 to 17.88)	2.22 (−4.25 to 8.68)	0.96 (0.63 to 1.45)	1.16 (0.64 to 2.10)
	Non-Hispanic other	6.83 (0.18 to 13.48)	6.59 (−0.57 to 13.75)	0.60 (0.38 to 0.93)	1.88 (1.03 to 3.46)
**Education**					
	Less than high school	13.40 (1.93 to 24.88)	−7.03 (−22.16 to 8.10)	0.52 (0.24 to 1.11)	2.22 (0.66 to 7.47)
	High school or General Educational Development degree	3.15 (−1.31 to 7.61)	0.62 (−4.66 to 5.90)	1.12 (0.82 to 1.52)	1.33 (0.79 to 2.25)
	Some college	−5.65 (−9.83 to −1.48)	−2.64 (−6.99 to 1.71)	0.89 (0.67 to 1.19)	1.60 (1.05 to 2.45)
	College degree or greater education	Reference	Reference	Reference	Reference

#### Tobacco Product Purchases

There were no significant differences in the odds of purchasing tobacco products between participants exposed to graphic (current smokers: OR 0.93, 95% CI 0.67 to 1.29; *P*=.66 and recent quitters: OR 0.73, 95% CI 0.44 to 1.19; *P*=.20) or supportive (current smokers: OR 1.05, 95% CI 0.75 to 1.46; *P*=.78 and recent quitters: OR 0.73, 95% CI 0.43 to 1.18; *P*=.20) ads versus those not exposed to antismoking ads (Table 3). Similarly, there was no difference in tobacco purchases by presence of price promotions (current smokers: OR 1.09, 95% CI 0.86 to 1.38; *P*=.49 and recent quitters: OR 0.90, 95% CI 0.62 to 1.31; *P*=.60; Table 3).

## Discussion

### Principal Findings

In a virtual convenience store shopping experiment conducted with adults, this analysis found a lower urge to smoke among current smokers who viewed supportive antismoking ads than those who did not view antismoking ads. On average, participants exposed to supportive antismoking ads reported urges to smoke that were 5 points lower (or 5% lower as the scale was 0-100) than those of participants not exposed to antismoking ads. This finding has important implications for the potential benefits of supportive antismoking ads at the POS. Although the direction of the effect was consistent with the results for supportive ads among current smokers, we found no effect of supportive antismoking ads for recent quitters (quit smoking in the past year) or for graphic antismoking ads regardless of smoking status. We also tested whether purchasing 1 or more tobacco products while in the virtual store varied by exposure to antismoking ads or price promotions among current smokers and recent quitters. However, none of these relationships were significant.

### Importance of Findings

Our finding of a lower urge to smoke among current smokers exposed to supportive ads suggests that supportive ads may be a method of decreasing urges to smoke among cigarette smokers. Urges to smoke are an important determinant of actual smoking behavior among current smokers [28] and self-efficacy to quit smoking among smokers attempting to quit smoking [29].

### Comparison With Prior Work

Regarding our specific finding of the relationship between urge to smoke and supportive antiads, we were unable to find any previous study that examined this relationship in a virtual convenience store.

In terms of graphic ads, 1 other study [17] has examined variation in urge to smoke by exposure to graphic ads in a virtual convenience store and found results consistent to ours. Using a different convenience sample of adult current smokers and recent quitters than this analysis, Kim et al [17] examined urge to smoke while exposing participants to an open (standard) or enclosed tobacco display in iShoppe. Kim et al [17] found no difference in urge to smoke between participants exposed to a graphic antismoking ad versus no antismoking ad on the enclosed display.

Regarding price promotions, we were able to locate 1 study that examined the relationship between exposure to POS advertising, including tobacco promotions such as special prices, multipack discounts, or free gifts with purchase of cigarettes, and cravings to smoke [30]. Siahpush et al [6] found a positive relationship between promotions and cravings to smoke that approached significance (*P*=.06). Our findings for price promotions did not approach this level of significance; however, our experimental design and analysis also included antismoking ads, which Siahpush et al [6] did not include.

Unfortunately, we were unable to find any existing research that examined the relationship between exposure to price promotions and tobacco purchases. The only related study examined the relationship between the use of price promotions when making a tobacco purchase and the size of the purchase [14]. Despite the lack of research on the impact of price promotions on purchases in the United States, bans on price promotions have been cited as one of the most effective tobacco control efforts in Europe [19].

### Limitations

Several limitations apply to this analysis. Owing to our use of a convenience sample, the results of this analysis may not generalize to all current cigarette smokers and recent quitters in the United States. However, specific aspects of our experimental design, such as randomization and adjusting for covariates, created comparable experimental conditions and, thereby, minimized threats to internal validity. After participants were divided into multiple experimental conditions, our sample sizes were rather small. However, collapsing the purchasable ad space only and high-visibility conditions improved sample sizes somewhat. In addition, it remains unclear whether the results of this experiment can be generalized to behavior in brick-and-mortar convenience stores or to Web purchases. However, existing research has found similarities between virtual store purchases and real-life purchases [31-34]. In addition, because we only used antismoking ads from *Tips*, the results of this analysis may not generalize to all antismoking ads. Finally, it is possible that some participants in the virtual store experiment did not notice the ads. However, because the study focused on antismoking ad exposure, we could not also adjust for awareness of the antismoking ads because awareness only varies among participants who have viewed the ads.

### Conclusions

This analysis supports the potential utility of future research on the ability of supportive antismoking ads to combat urges to smoke among current cigarette smokers. Given that urge to smoke is an important predictor of smoking behavior, research should continue to explore the utility of antismoking ads as a method of influencing tobacco purchases at the POS. Given the existing research suggesting that the context and type of antismoking ads in stores can affect attention to and reactions to antismoking ads [35,36], if antismoking ads are used, the choice and placement of antismoking ads should be carefully considered when using these ads as a tobacco control intervention.

## Appendix

Multimedia Appendix 1 Table depicting the partial cross-over randomized design that resulted in 10 experimental groups.

Multimedia Appendix 2 Images of the experimental conditions.

Multimedia Appendix 3 Flow diagram demonstrating how participants were assigned to 10 experimental conditions and then condensed into 6 conditions for the analysis.

## Abbreviations

3D: 3-dimensional
CDC: Centers for Disease Control and Prevention
e-cigarettes: electronic cigarettes
OR: odds ratio
POS: point of sale

## References

[1] Surgeon General, US (2014). The Health Consequences of Smoking--50 Years of Progress: A Report of the Surgeon General.

[2] (2017). Federal Trade Commission.

[3] Mackintosh AM, Moodie C, Hastings G (2012). The association between point-of-sale displays and youth smoking susceptibility. Nicotine Tob Res.

[4] Kim AE, Loomis BR, Busey AH, Farrelly MC, Willett JG, Juster HR (2013). Influence of retail cigarette advertising, price promotions, and retailer compliance on youth smoking-related attitudes and behaviors. J Public Health Manag Pract.

[5] Germain D, McCarthy M, Wakefield M (2010). Smoker sensitivity to retail tobacco displays and quitting: a cohort study. Addiction.

[6] Siahpush M, Shaikh RA, Smith D, Hyland A, Cummings KM, Kessler AS, Dodd MD, Carlson L, Meza J, Wakefield M (2016). The association of exposure to point-of-sale tobacco marketing with quit attempt and quit success: results from a prospective study of smokers in the United States. Int J Environ Res Public Health.

[7] Martino SC, Setodji CM, Dunbar MS, Shadel WG (2019). Increased attention to the tobacco power wall predicts increased smoking risk among adolescents. Addict Behav.

[8] Xu X, Wang X, Caraballo RS (2016). Is every smoker interested in price promotions? An evaluation of price-related discounts by cigarette brands. J Public Health Manag Pract.

[9] Coady MH, Chan CA, Auer K, Farley SM, Kilgore EA, Kansagra SM (2013). Awareness and impact of New York City's graphic point-of-sale tobacco health warning signs. Tob Control.

[10] Clattenburg EJ, Elf JL, Apelberg BJ (2013). Unplanned cigarette purchases and tobacco point of sale advertising: a potential barrier to smoking cessation. Tob Control.

[11] Carter OB, Mills BW, Donovan RJ (2009). The effect of retail cigarette pack displays on unplanned purchases: results from immediate postpurchase interviews. Tob Control.

[12] Wakefield M, Germain D, Henriksen L (2008). The effect of retail cigarette pack displays on impulse purchase. Addiction.

[13] Ross H, Tesche J, Vellios N (2017). Undermining government tax policies: common legal strategies employed by the tobacco industry in response to tobacco tax increases. Prev Med.

[14] Doogan NJ, Cooper S, Quisenberry AJ, Brasky TM, Browning CR, Klein EG, Hinton A, Nagaraja HN, Xi W, Wewers ME (2018). The role of travel distance and price promotions in tobacco product purchase quantity. Health Place.

[15] Durkin SJ, Biener L, Wakefield MA (2009). Effects of different types of antismoking ads on reducing disparities in smoking cessation among socioeconomic subgroups. Am J Public Health.

[16] Lin PN, Zimmermann MH, Manderski MT, Schmelzer AC, Steinberg MB (2012). Evaluation of graphic cigarette warning images on cravings to smoke. J Smok Cessat.

[17] Kim AE, Nonnemaker JM, Loomis BR, Shafer PR, Shaikh A, Hill E, Holloway JW, Farrelly MC (2014). Influence of point-of-sale tobacco displays and graphic health warning signs on adults: evidence from a virtual store experimental study. Am J Public Health.

[18] (2018). Food and Drug Administration.

[19] Schaap MM, Kunst AE, Leinsalu M, Regidor E, Ekholm O, Dzurova D, Helmert U, Klumbiene J, Santana P, Mackenbach JP (2008). Effect of nationwide tobacco control policies on smoking cessation in high and low educated groups in 18 European countries. Tob Control.

[20] (2017). Truth Initiative.

[21] Yang D (2013). The communication effects of audience situation and message framing on smoking cessation. Soc Sci Res Netw.

[22] Schneider TR, Salovey P, Pallonen U, Mundorf N, Smith NF, Steward WT (2001). Visual and auditory message framing effects on tobacco smoking. J Appl Social Pyschol.

[23] Sutfin EL, Szykman LR, Moore MC (2008). Adolescents' responses to anti-tobacco advertising: exploring the role of adolescents' smoking status and advertisement theme. J Health Commun.

[24] Kim AE, Nonnemaker JM, Loomis BR, Baig A, Hill E, Holloway JW, Farrelly MC, Shafer PR (2013). Influence of tobacco displays and ads on youth: a virtual store experiment. Pediatrics.

[25] Guillory J, Kim AE, Nonnemaker JM, Bradfield B, Taylor NH, Dutra L, Feld A (2019). Effect of menthol cigarette and other menthol tobacco product bans on tobacco purchases in the RTI iShoppe virtual convenience store. Tob Control.

[26] Dutra LM, Nonnemaker J, Taylor N, Kim AE (2018). Deception and shopping behavior among current cigarette smokers: a web-based, randomized virtual shopping experiment. JMIR Res Protoc.

[27] Bursac Z, Gauss CH, Williams DK, Hosmer DW (2008). Purposeful selection of variables in logistic regression. Source Code Biol Med.

[28] Shiffman S, Dunbar M, Kirchner T, Li X, Tindle H, Anderson S, Scholl S (2013). Smoker reactivity to cues: effects on craving and on smoking behavior. J Abnorm Psychol.

[29] Niaura R, Shadel WG, Britt DM, Abrams DB (2002). Response to social stress, urge to smoke, and smoking cessation. Addict Behav.

[30] Siahpush M, Shaikh RA, Cummings KM, Hyland A, Dodd M, Carlson L, Kessler AS, Meza J, Wan N, Wakefield M (2016). The association of point-of-sale cigarette marketing with cravings to smoke: results from a cross-sectional population-based study. Tob Control.

[31] Aubin G, Béliveau MF, Klinger E (2018). An exploration of the ecological validity of the virtual action planning-supermarket (VAP-S) with people with schizophrenia. Neuropsychol Rehabil.

[32] Desmet P, Bordenave R, Traynor J (2013). Differences in purchasing behaviour between physical and virtual laboratory stores. Recherche et Applications en Marketing.

[33] van Herpen E, van den Broek E, van Trijp HC, Yu T (2014). Library Wageningen University & Research.

[34] Waterlander WE, Jiang Y, Steenhuis IH, Mhurchu CN (2015). Using a 3D virtual supermarket to measure food purchase behavior: a validation study. J Med Internet Res.

[35] Dutra LM, Nonnemaker J, Guillory J, Bradfield B, Taylor N, Kim A (2018). Smokers' attention to point-of-sale anti-smoking ads: an eye-tracking study. Tob Regul Sci.

[36] Kim A, Nonnemaker J, Guillory J, Shafer P, Parvanta S, Holloway J, Farrelly M (2017). Antismoking ads at the point of sale: the influence of ad type and context on ad reactions. J Health Commun.

