# Supplementary material for: Antismoking Advertisements and Price Promotions and Their Association With the Urge to Smoke and Purchases in a Virtual Convenience Store: Randomized Experiment
Source: J Med Internet Res. 2019 Oct 23;21(10):e14143. doi: 10.2196/14143 (PMC6914233; doi:10.2196/14143)
Supplement: Multimedia Appendix 1 [file jmir_v21i10e14143_app1.pdf]

Table 1. Experimental conditions in the RTI iShoppeTM virtual store

	Price promotion ban	
Anti-Smoking Ads (Type and placement)	**Promotions banned**	**Promotions present**
**None**	1	2
**Graphic ads in purchasable ad space**	3	4
**Graphic ads in purchasable and high visibility ad space**	5	6
**Supportive ads in purchasable ad space**	7	8
**Supportive ads in purchasable and high visibility ad space**	9	10
