# Supplementary material for: Antismoking Advertisements and Price Promotions and Their Association With the Urge to Smoke and Purchases in a Virtual Convenience Store: Randomized Experiment
Source: J Med Internet Res. 2019 Oct 23;21(10):e14143. doi: 10.2196/14143 (PMC6914233; doi:10.2196/14143)

Figure 1. Condition 1: Anti-smoking ads are absent, and price promotions are banned.

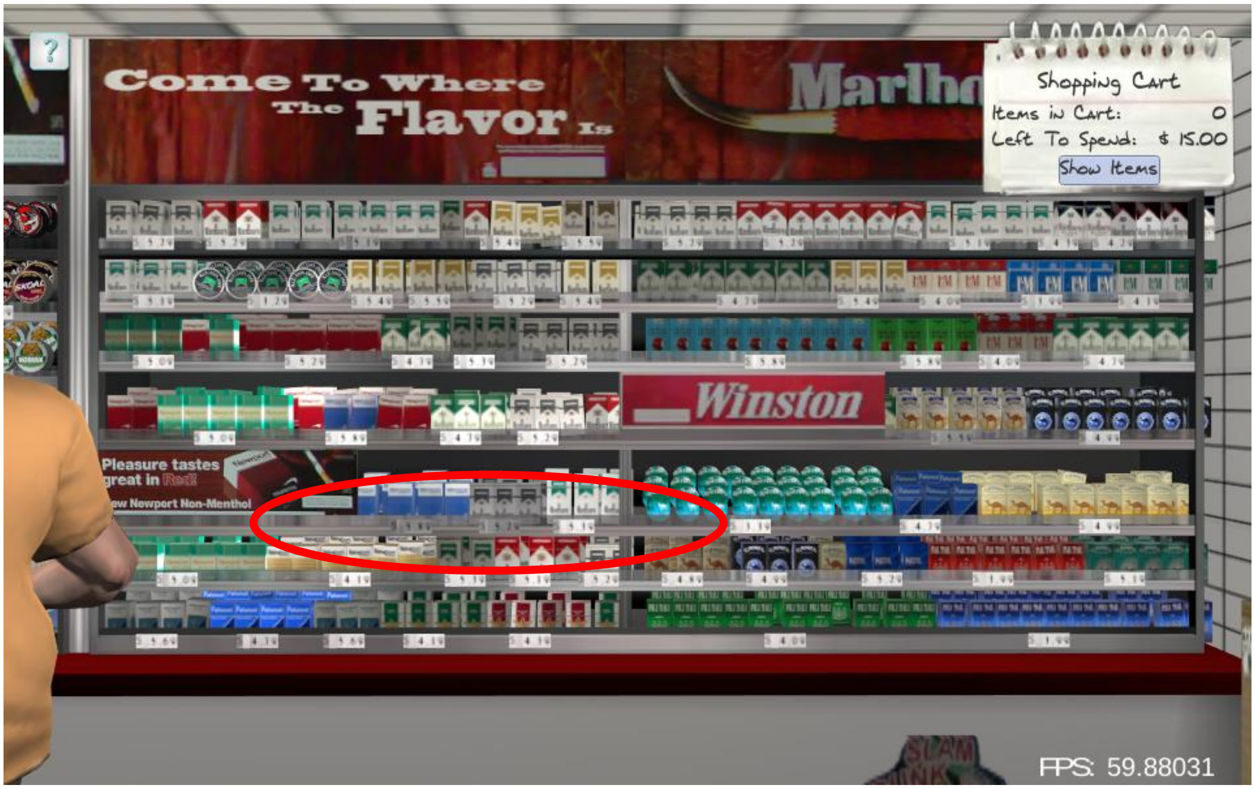

Figure 2. Condition 2: Anti-ads are absent, and price promotions are present.

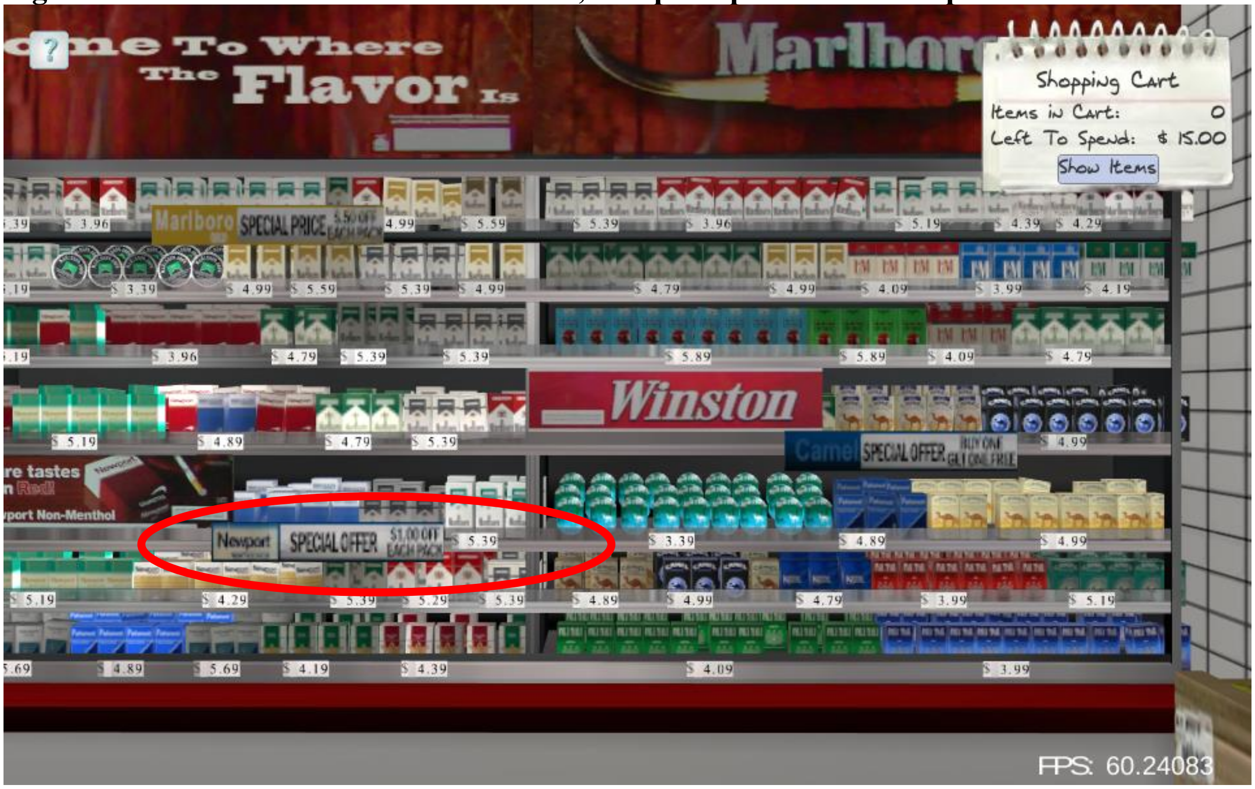

Figure 3. Condition 3: Graphic ads are present in purchasable ad space (e.g., interior and exterior windows, gas pump topper), and price promotions are banned.

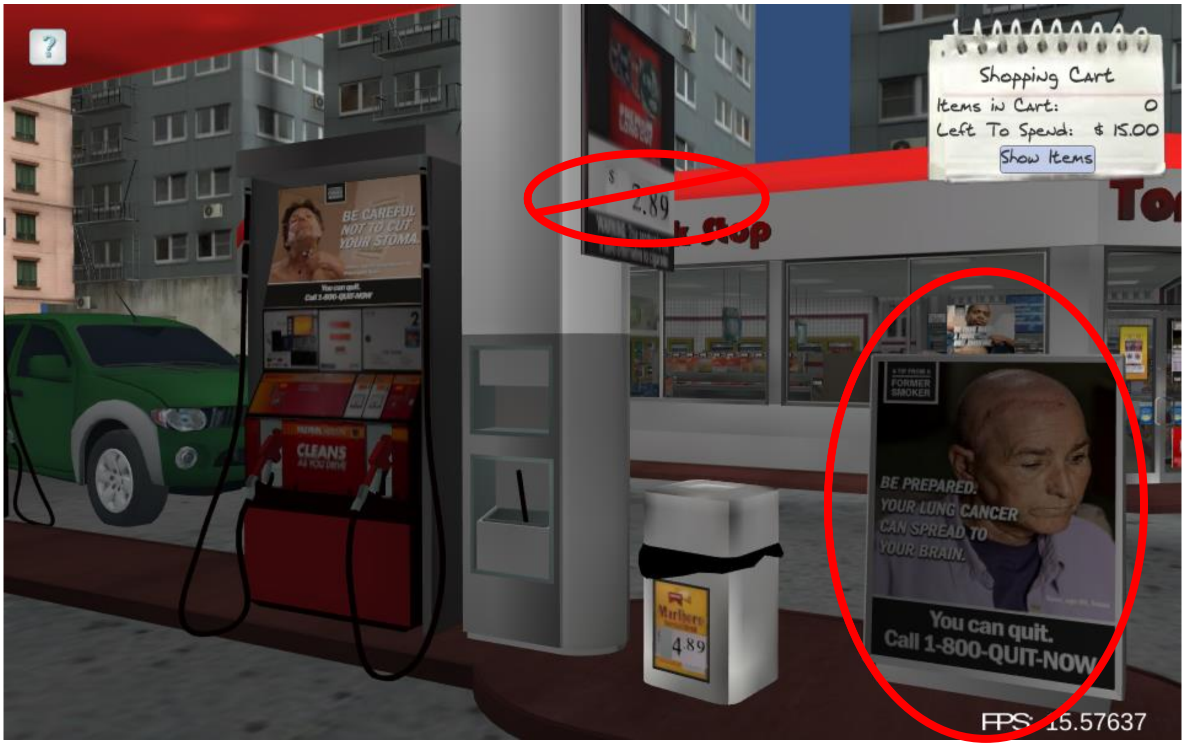

Figure 4. Condition 4: Graphic ads are present in purchasable ad space (e.g., interior and exterior windows, gas pump topper), and price promotions are present (circled text reads "SPECIAL OFFER: Buy two packs, get one free").

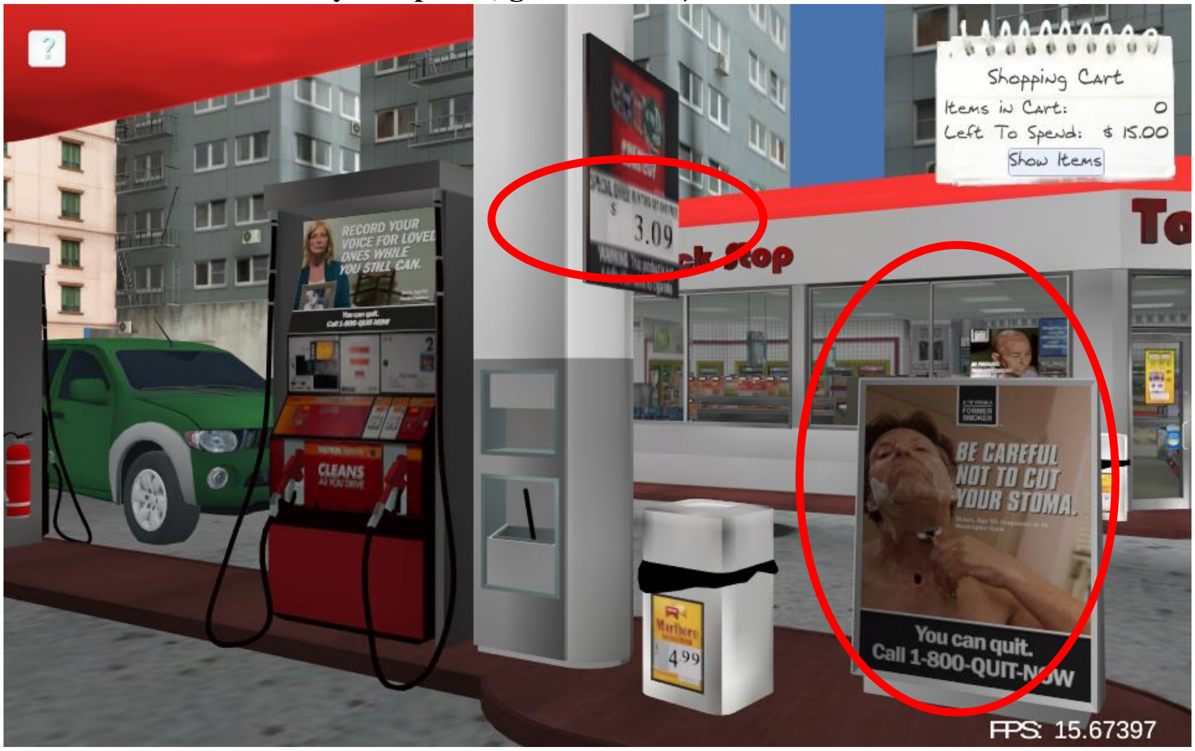

Figure 5. Condition 5: Graphic ads are placed in purchasable (e.g., interior and exterior windows, gas pump topper) and high visibility (e.g., by checkout counter, hanging from the ceiling between aisles) ad space, and price promotions are banned.

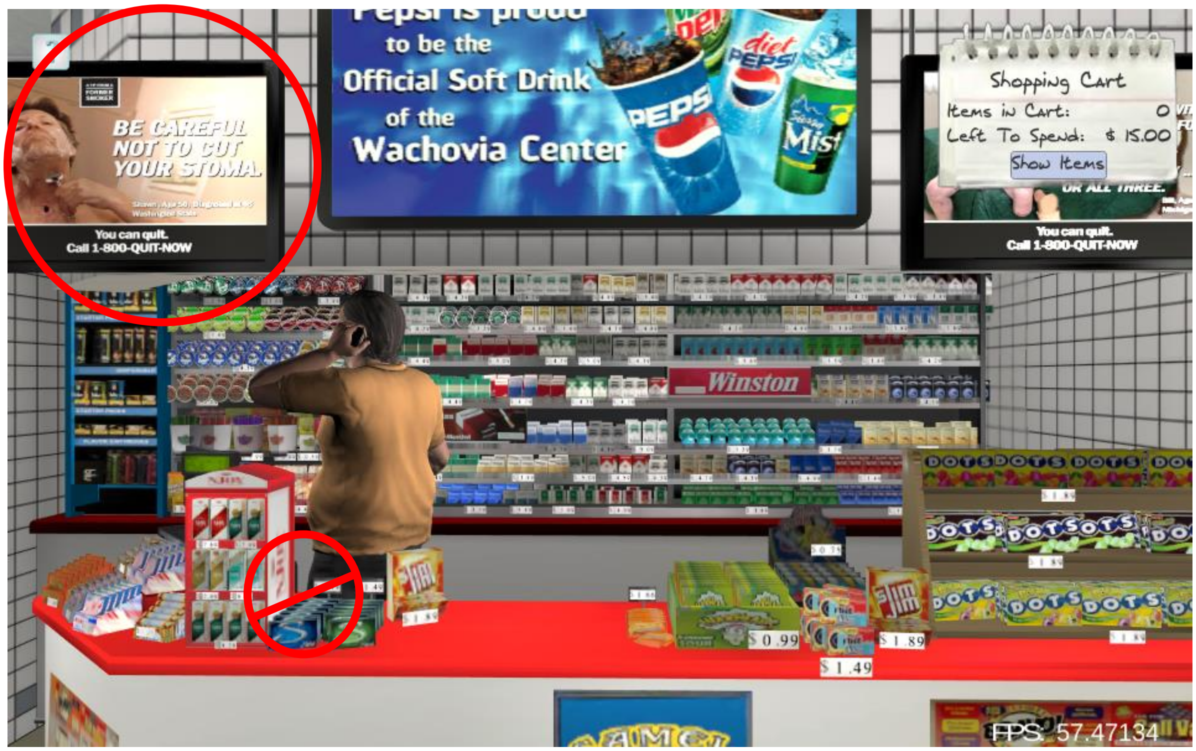

Figure 6. Condition 6: Graphic ads are placed in purchasable (e.g., interior and exterior windows, gas pump topper) and high visibility (e.g., by checkout counter, hanging from the ceiling between aisles) ad space, and price promotions are present (circled text reads "Buy One Get One Free").

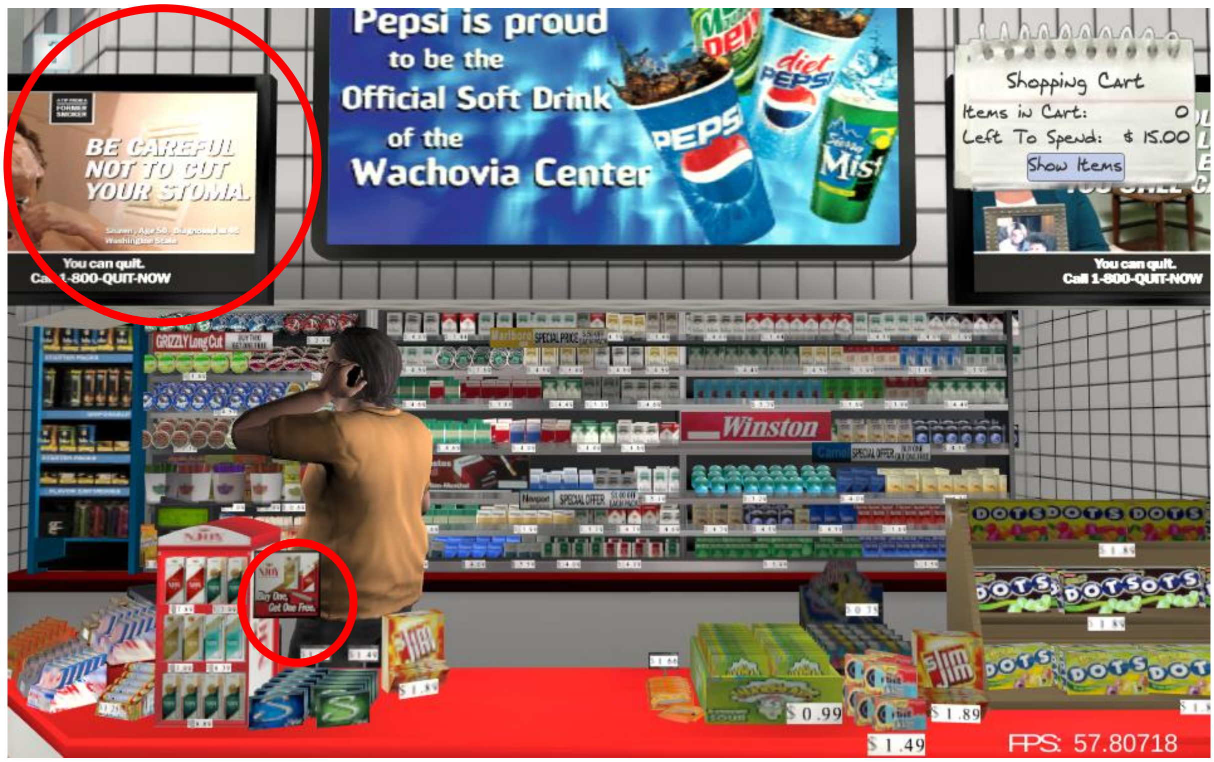

Figure 7. Condition 7: Supportive ads are placed in (e.g., interior and exterior windows, gas pump topper) ad space, and price promotions are banned.

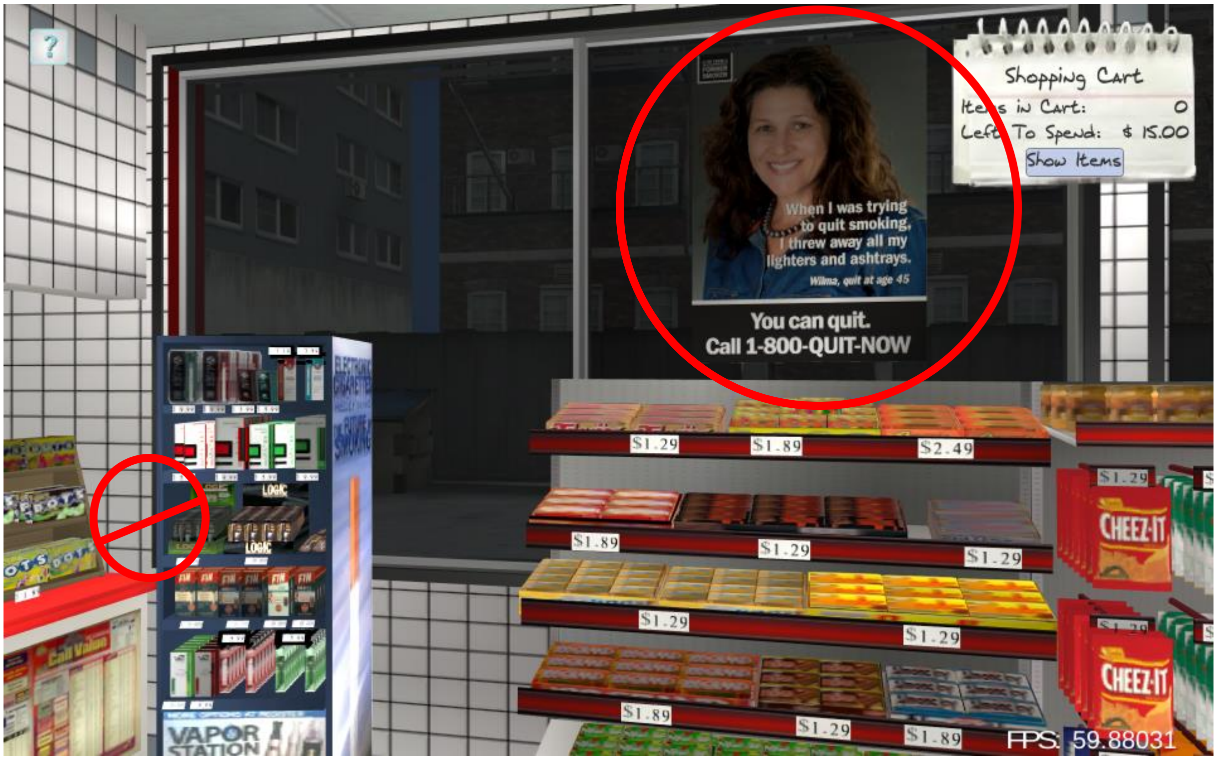

Figure 8. Condition 8: Supportive ads are placed in purchasable ad space (e.g., interior and exterior windows, gas pump topper), and price promotions are present (text reads “SPECIAL PRICE $1 OFF”).

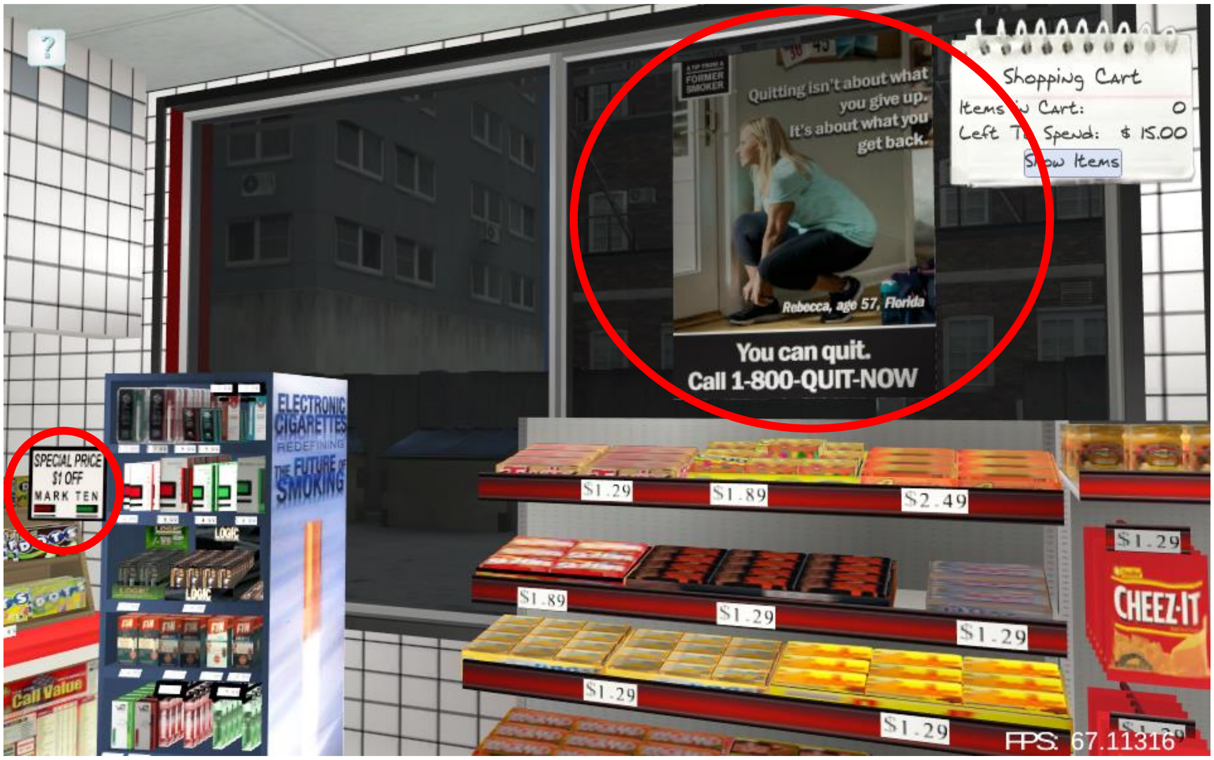

Figure 9. Condition 9: Supportive ads are placed in in purchasable (e.g., interior and exterior windows, gas pump topper) and high visibility (e.g., by checkout counter, hanging from the ceiling between aisles) ad space, and price promotions are banned.

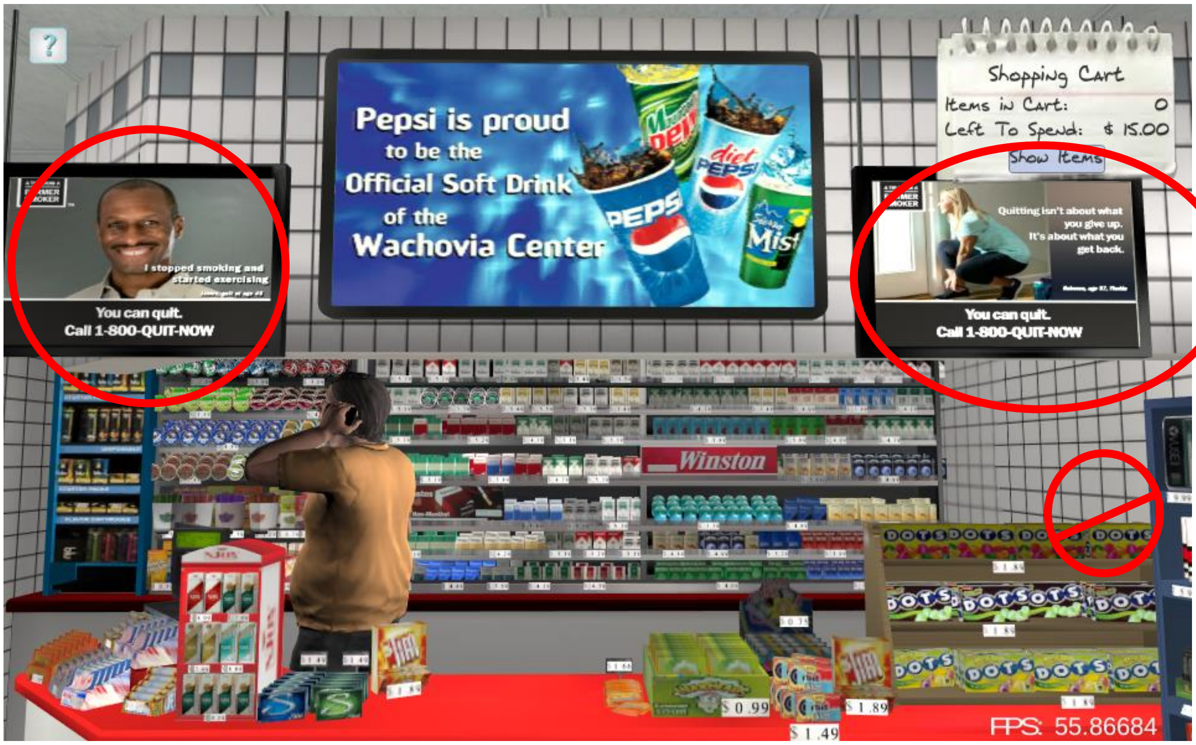

Figure 10. Condition 10: Supportive ads are placed in in purchasable (e.g., interior and exterior windows, gas pump topper) and high visibility (e.g., by checkout counter, hanging from the ceiling between aisles) ad space, and price promotions are present.

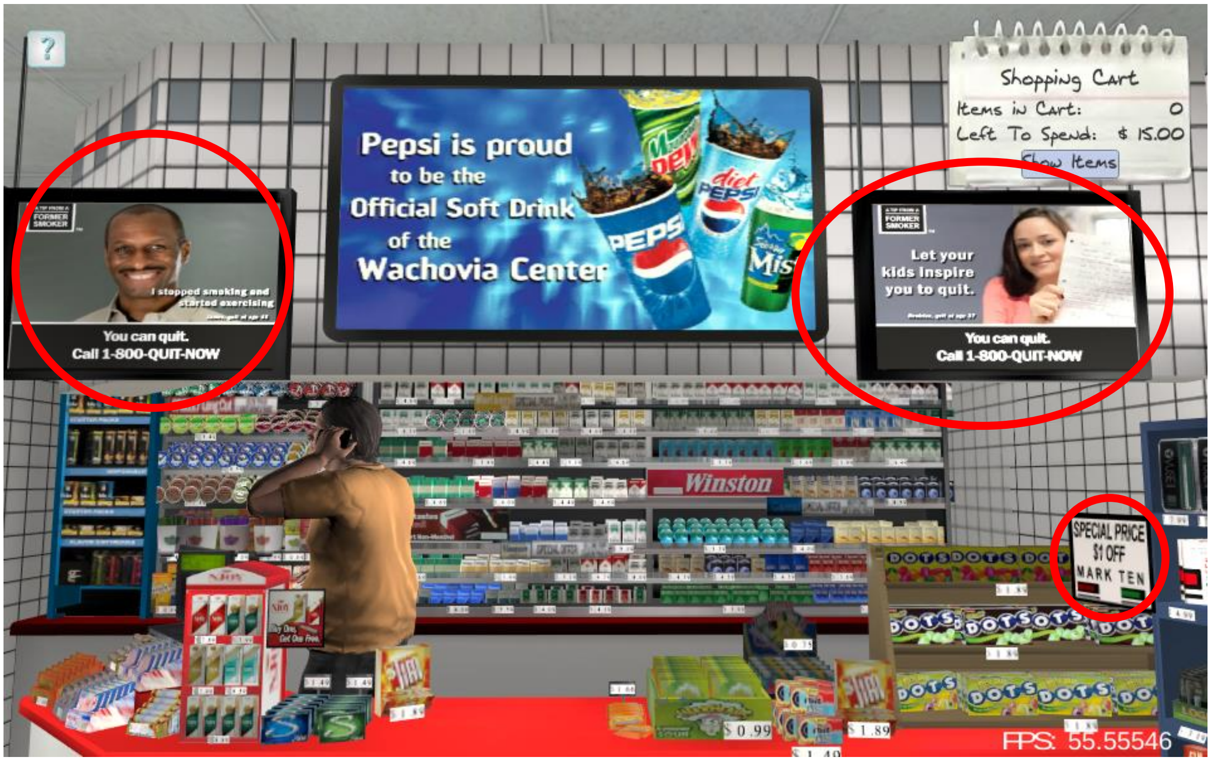

Supplement: Multimedia Appendix 2 [file jmir_v21i10e14143_app2.pdf]
