# Supplementary figures and images for: Antismoking Advertisements and Price Promotions and Their Association With the Urge to Smoke and Purchases in a Virtual Convenience Store: Randomized Experiment
Source: J Med Internet Res. 2019 Oct 23;21(10):e14143. doi: 10.2196/14143 (PMC6914233; doi:10.2196/14143)

Figure 1. Flow diagram for experimental conditions in iShoppe™ experiment

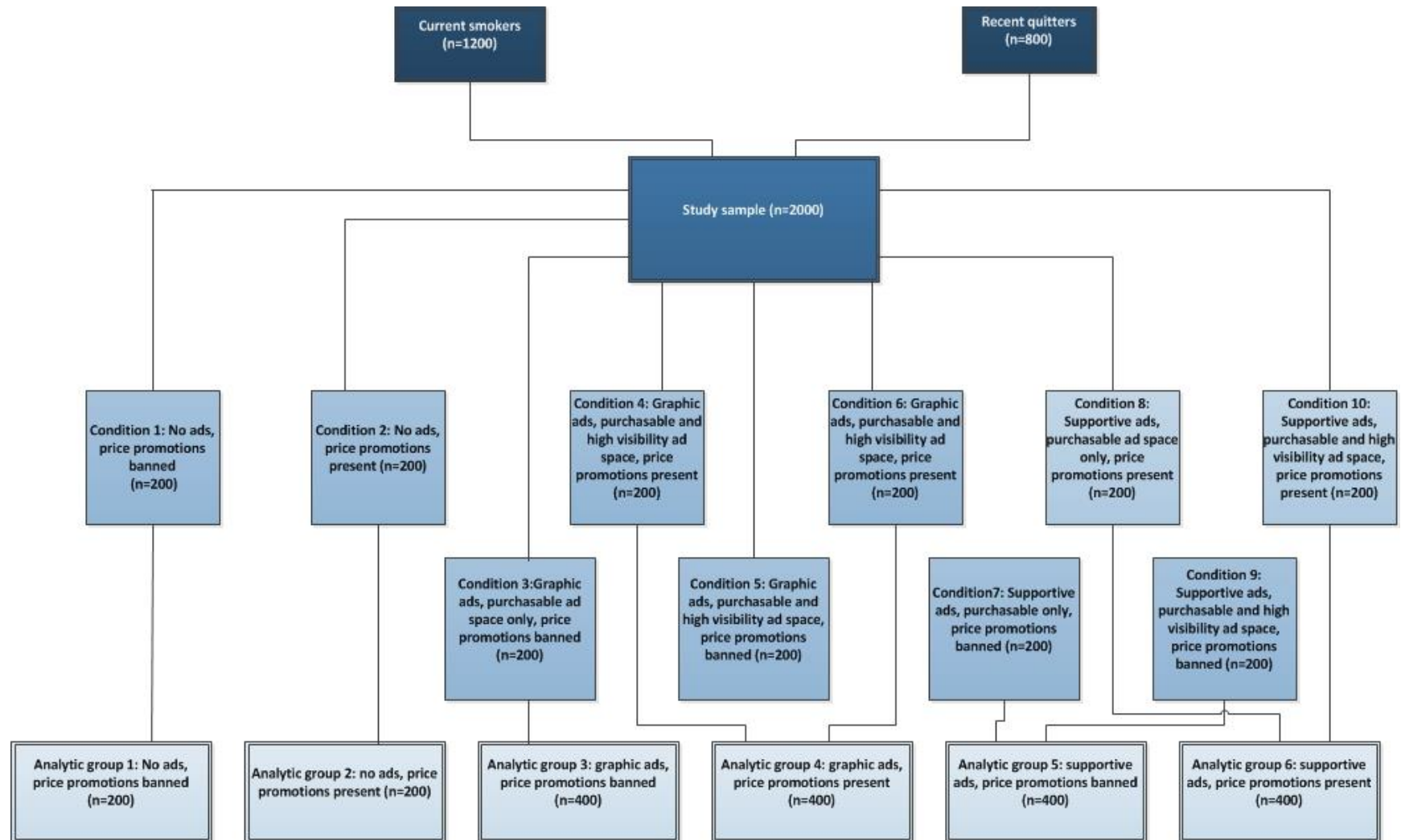

Supplement: Multimedia Appendix 3 [file jmir_v21i10e14143_app3.pdf]
